# Use of DSLR and Sonic Cameras to Detect and Locate High-Voltage Corona Discharges

**DOI:** 10.3390/s22197250

**Published:** 2022-09-24

**Authors:** Jordi-Roger Riba, Pau Bas-Calopa

**Affiliations:** Electrical Engineering Department, Universitat Politècnica de Catalunya, Rambla Sant Nebridi 22, 08222 Terrassa, Spain

**Keywords:** partial discharges, corona discharges, high-voltage, optic methods, acoustic methods

## Abstract

Corona discharges are a concern in high-voltage applications. It is of utmost importance to detect and locate the discharges at an early stage using simple methods for this purpose. This paper evaluates and compares the sensitivity of two methods for detecting and locating the source of discharges, which are based on a digital single-lens reflex (DSLR) camera and a portable wideband sonic camera incorporating a matrix of micro-electromechanical systems (MEMS) microphones. Both cameras can generate an image of the studied area where the discharge sites are identified. The study is carried out with different electrode geometries, 50 Hz alternating current (ac) and positive and negative direct current (dc) supplies, and the effect of the distance between the sensor and the discharge sites is also analyzed. The presented results show that the sonic camera enables fast, simple, and sensitive detection and localization of the source of corona discharges even at a very early stage in daylight conditions, regardless of the type of power supply, that is, ac or positive/negative dc, and at distance of several meters from the discharge source.

## 1. Introduction

Electrical discharges are typical of insulation systems in high-voltage applications, due to the continuous effects of the high electric stress. According to the IEC 60270 international standard [1], partial discharges (PDs) are localized electrical discharges that only partially bridge the insulation between two electrodes. PDs are typical of high-voltage applications, although they can also appear in medium-voltage and even in low-voltage applications [2] incorporating fast-switching semiconductors and/or inverter-fed loads, where the affected elements are motors [3] and cable systems [2] among others.

PDs are generated at inhomogeneous sites of solid, liquid, or gaseous insulation systems [4]; at surfaces and/or interfaces; or between a conductor and a floating metallic element [5]. PDs produce premature aging of organic insulation systems [6,7,8], as they cause thermal, mechanical, chemical, and structural [9] changes, as well as environmental aging [10]. Therefore, PD activity must be detected and the PD sources identified in order to apply corrective actions before significant insulation damage occurs [11]. PDs are well known to generate ultraviolet and visible light emissions, audible noise (AN), a local temperature rise, chemicals, and also current pulses. [7]. Therefore, PDs are commonly detected by electromagnetic methods such as radio interference voltage and PD detectors [12], UHF and VHF antennas [13], acoustic [14] and ultrasonic sensors [15], optical [16,17] and infrared detectors [15,18], or X-ray detectors [19,20], among others. However, only specific detection methods allow the direct location of discharge sites.

PDs that are produced in a gaseous medium are known as corona discharges. Corona is only produced in highly inhomogeneous electric fields [21], and is generated at voltages that are lower than those that are required for complete breakdown [22]. Corona discharges locally ionize the gaseous insulation surrounding an energized electrode when the electric field strength exceeds a critical inception value [23,24]. It is worth noting that in low pressure environments, PDs and corona activity appear at lower voltage levels than required at standard pressure [25]. Similar to PDs, corona discharges generate ultraviolet and visible light [12,26,27], electromagnetic radiation, audible noise (AN) [28] and different chemical components such as ozone [29] or NOx compounds [30], among others. Due to the low level of visible radiation that is emitted, corona is very difficult to detect in daylight conditions, so high-voltage laboratories typically detect corona discharges in the dark [24] using high-performance digital cameras [31]. 

Due to the impact of electrical discharges in insulation systems, there are different international standards to detect their effects based on the detection of PDs (IEC 60270:2000), the detection of radio interference voltage (NEMA 107-2016), or the detection of visual corona and RIV (IEEE Std. 1829-2017, or the ANSI/NEMA CC1-2009 which is specific for substation connectors).

Different recent research works have analyzed the behavior of a commercial sonic camera that is composed of a matrix of microphones, although early works [32,33] proposed the use of microphone arrays for the detection of electrical discharges. In [34] the sonic camera was used to detect PDs in power-line elements, concluding that it is able to detect different failures and their severity when operated by technicians with minimal experience. A similar paper [35] compared the behavior of the sonic camera against a PD detector, concluding that the sonic camera is easier to operate and the most suitable for on-site and on-line testing, although less accurate than the PD detector. However, an accurate sensitivity comparison that is based on the PD inception voltage value was not performed. In [36] it was shown that the sonic camera is also capable of detecting discharges with dc supply.

In [37], the performance of a sonic camera was compared to that of a dual-band UV camera, concluding that both cameras have similar sensitivity, but dc discharges were not analyzed. In [38] it was concluded that the sonic camera is effective in detecting external defects when there is a clear line of sight.

Different types of sensors are used to detect corona discharges, including radio interference voltage and PD detectors and antennas to detect the electromagnetic emissions that are associated with corona, or acoustic sensors. However they are often too expensive, complex, or difficult to use in field applications; are affected by electromagnetic noise; or do not allow direct and easy location of discharge points [27]. Due to such limitations, this paper compares the sensitivity of two types of hand-held cameras, that is, a digital single-lens reflex camera (DSLR) and a sonic camera, which allow corona discharge points to be located on-site. Both cameras include a sensor consisting of a matrix of sensing elements, thus generating an image that allows locating the discharge sites. For this purpose, we find the minimum voltage at which corona is detected for both cameras at different distances between the discharge site and the sensors in the range of 1 m to 10 m. To the knowledge of the authors, there are no scientific works performing such a comparison. This work is contributing to the area of location of corona discharges by using an acoustic method, since it allows on-site and on-line detection and identification of electrical discharges with minimal operator experience. The topics that were analyzed in this paper have a great potential for application in the field of predictive maintenance of high-, medium-, and low-voltage electric systems. 

The article is organized in the following manner. Section 2 describes the characteristics of the analyzed sonic and DSLR cameras. Section 3 describes the experimental setup to generate the corona discharges and to compare the behavior of both cameras. Section 4 develops and discusses the experimental results. Finally, Section 5 concludes this experimental paper.

## 2. The Analyzed Sonic and DSLR Cameras

As already explained, this paper compares the sensitivity of a sonic and a DSLR camera to detect and locate corona discharges. While digital DSLR cameras are well known and were introduced to the market over 30 years ago, the sonic camera that is discussed in this paper is fairly new.

In a previous study [24], the authors of this work showed that in an unscreened laboratory, the DSLR camera that was analyzed here can provide the same sensitivity as a high performance PD detector. In this test a PD instrument (PD-BaseII, Techimp, Zola Predosa, Bolognia, Italy) was applied using the standard IEC 60270 [1] bandwidth (115–440 kHz) and a sampling frequency of 200 M samples/s, which gives the measured voltage as equivalent charge expressed in pC. The PD detector was calibrated using a PD calibrator (PDCAL Techimp, Zola Predosa, Bolognia, Italy) using the average value of 2000 calibration pulses to minimize the effect of the background noise. For this reason, the DSLR camera is taken as a reference for its proven high sensitivity.

### 2.1. The DSLR Camera

The wavelengths of light characteristic of corona discharges lie primarily within the boundary between the UV and visible regions of the spectrum, regardless of the type of power supply, that is, ac or positive/negative dc [39]. Therefore, digital cameras that are equipped with CMOS sensors are useful for this purpose, since they are sensitive to said wavelength range and have attractive features such as reduced power consumption and low voltage supply [40], or excellent quantum efficiency in the visible range, although more limited in the UV interval [41]. Optical sensors are more advantageous than other sensing methods because they are immune to background electromagnetic noise that is typical of power systems. Digital image sensors offer a simple solution for locating discharge sites [42], thus potentially allowing corona discharges to be detected and located at an early stage. Commercial image sensors include a matrix with a large number of photosensitive elements, typically photodiodes, which generate charge in response to incident light. Photoelectrons are generated when light photons of sufficient energy impact the image sensor. The number of photoelectrons that are generated is related to the intensity of the incident light, which allows the image to be reconstructed [43] by detecting the near-UV and visible photons that are produced during the corona discharge process [7] as a consequence of ionization, excitation, and recombination processes [44]. Digital cameras are known to detect both the near UV and the visible spectrum [45]. 

DSLR cameras make it possible to detect corona activity using a long exposure mode when the background is darker than the corona light [46]. A Canon EOS-70D digital single-lens reflex camera (DSLR) was used to detect and locate the visual corona, the main characteristics of which are summarized in Table 1. Long exposure photographs were taken to detect light that was emitted by corona at the early stage, using a 60 s long exposition to capture more photons, f/5.6 aperture, ISO-800 sensitivity, and tungsten color temperature. 

**Figure 1:**
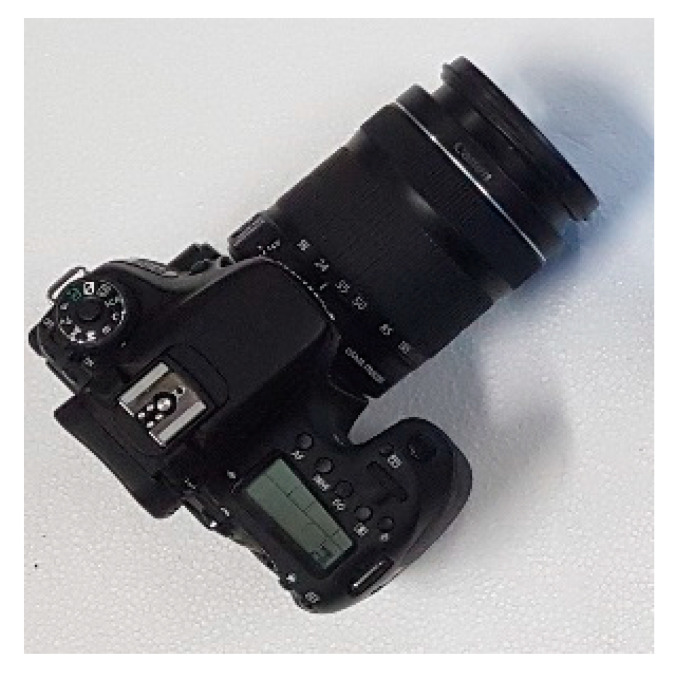
DSLR Canon EOS-70D camera.

Figure 1 shows the DSLR camera that was studied in this paper.

Although DSLR cameras are widely used in many applications, their use to detect PDs/corona is still limited, this paper contributing to this area. 

### 2.2. The Sonic Camera

When the electric field in the vicinity of an energized electrode that is surrounded by air exceeds an inception value, the air molecules become ionized [47], causing electrical discharges. Throughout the ionization process, the excited and released electrons change their energy levels, so that ultraviolet and visible photons are emitted, as well as broadband electromagnetic emissions, in addition to generating different chemicals including ozone (O_3_) or traces of nitric acid [48]. The ionization process also generates broadband audible noise (AN) in the range from one to several kilohertz [49] due to the rapid moving electrons colliding with neutral air molecules, with a sudden transfer of kinetic energy, which is equivalent to small explosions that are occurring at the corona sites, thus generating AN [50]. Therefore, corona discharges generate acoustic pulses. Under ac supply and near the corona inception voltage (CIV) value, the AN corona waveform consists of trains of periodic sound pressure pulses that appear close to the negative peaks of the ac voltage. When the voltage increases beyond the CIV value, the periodic sound pressure trains also appear near the positive peaks of the ac voltage waveform [51]. These pulses have harmonic components of 50 Hz. Under dc supply, due to the lack of any voltage modulation effect [50], no harmonic components are generated. Therefore, the AN that is induced by both negative and positive dc corona consists of several randomly distributed sound pressure pulses with broad frequency band. The amplitudes of such sound pulses are larger in the case of positive corona discharges [51]. In [52], it was found that the sound pressure pulses due to dc corona and the generated corona current pulses are correlated in the time domain, attributing this effect to the drift of the space charge that is generated by the corona discharges.

Acoustic methods are widely recognized as capable of detecting partial discharges, as described in the IEC TS 62748:2016 international standard [53]. They often use piezoelectric sensors jointly with suitable signal processing approaches [54,55]. For the detection and location of corona discharges, an NL sonic camera has been used, whose main characteristics are summarized in Table 2. The NL Camera is a device that is designed for the detection and localization of partial discharges in medium- and high-voltage electrical systems. It incorporates a standard digital camera combined with 124 MEMS microphones, which are well suited for applications in electrically noisy environments. The information that is provided by the matrix of microphones is superimposed on the visible image that is generated by the digital camera. In this way, the camera can detect and localize the audible emissions of partial discharges, allowing discharges to be detected at an early stage. The NL Camera instantly displays the location of the partial discharges on the screen, allowing effortless identification of their sources. For ac systems, it also displays the phase-resolved partial discharge pattern in real-time.

**Figure 2:**
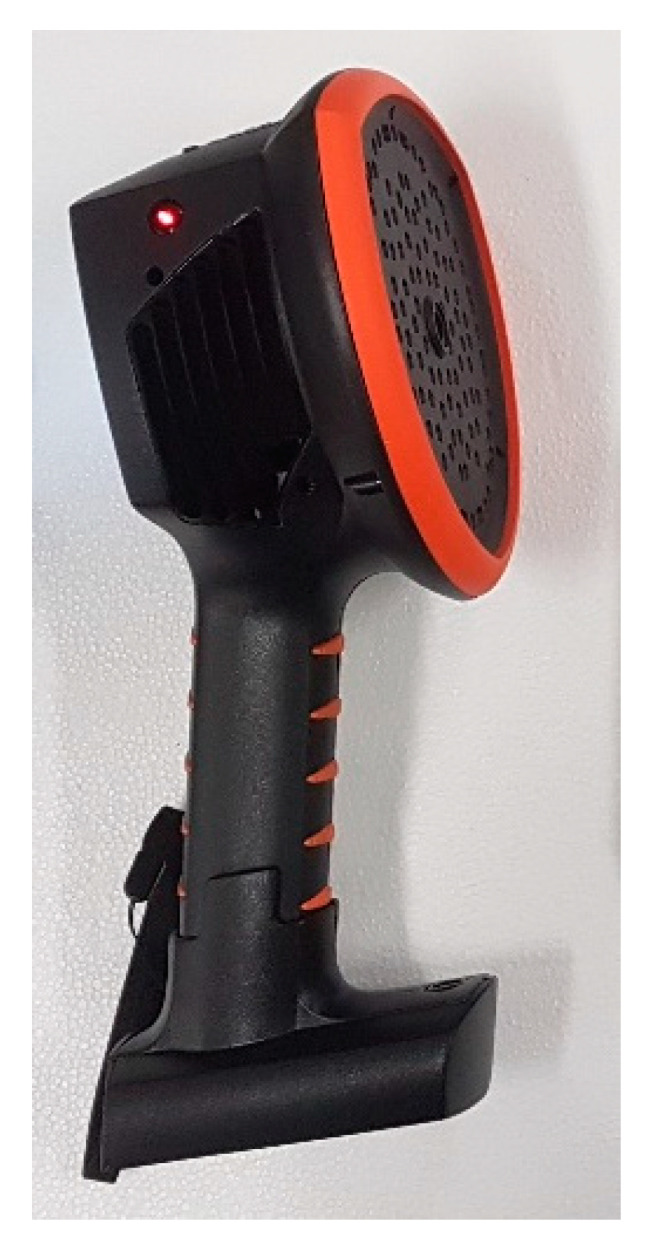
Sonic NL camera.

Figure 2 shows the sonic camera that was studied in this paper.

## 3. Experimental Setup

This section describes the experimental setup, the test objects that were used to generate the discharges, and the sensors for detecting and locating the partial discharges that were generated by different objects.

The corona tests that were analyzed in this work were carried out in the AMBER high-voltage laboratory of the Universitat Politècnica de Catalunya. The high-voltage was generated by means of a BK-130 ac hipot (0–130 kV, 50 Hz, 50 mA, Phenix Technologies, Accident, MD, USA) and two 4120-10 dc hipots (+120 kV, 10 mA; −120 kV, 10 mA, Phenix Technologies, Accident, MD, USA), which incorporate current and voltage measurement devices. 

**Figure 3:**
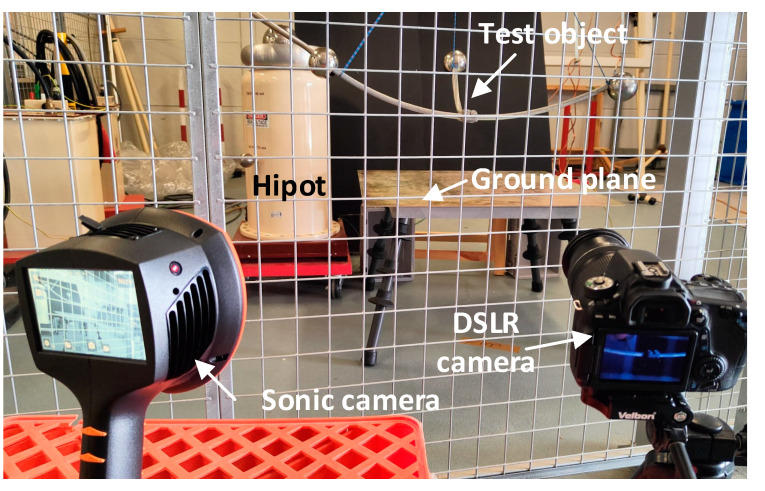
Experimental setup.

Figure 3 shows the experimental setup that was used to perform the tests, which includes different high-voltage generators (hipots), the test object where the corona discharges occur, and the DSLR and sonic cameras.

It is worth noting that the tests with the sonic camera were done in daylight conditions, while the tests with the DSLR camera were done in the dark.

### Analyzed Test Objects

**Figure 4:**
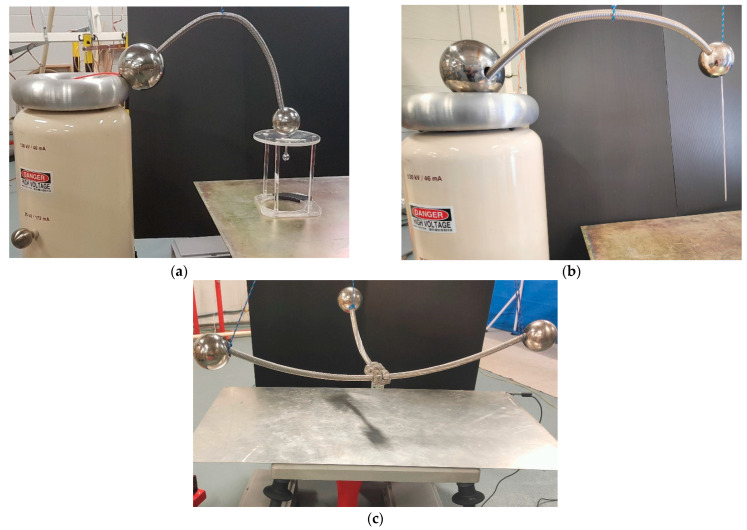
Test objects that were utilized to generate the corona discharges. (**a**) Sphere-plane gap (diameter = 22 mm, height above ground plane = 150 mm). (**b**) Needle-plane gap (curvature radius = 0.5 mm, slant angle = 12°, height above ground plane = 130 mm). (**c**) Substation connector (height above ground plane = 200 mm).

Different test objects were used to generate the corona discharges, including a sphere-plane air gap, a needle- plane air gap, and a substation connector, which are shown in Figure 4.

## 4. Experimental Results

This section summarizes the experimental results that were obtained with the three geometries that were analyzed (sphere-plane gap, needle-plane gap, and substation connector –plane gap) using the DSLR and sonic cameras. Experimental tests were conducted in an unscreened high-voltage indoor laboratory, at 19 °C, 43% relative humidity, and 990.3 hPa, therefore, the results that are presented are limited within the distance range of 0 to 10 m from the test object. 

It should be noted that the results that are presented in this section are based on the corona initiation voltage (CIV), i.e., the minimum voltage value at which corona activity is detected by progressively increasing the voltage from zero volts.

This paper applies the visible corona test method that was described in the IEEE 1829-2017 [56] guide. This standard clearly states that as the voltage increases, a negative polarity corona occurs before a positive corona, but the negative corona generates very little electromagnetic interference and acoustic noise. However, the inception of a positive polarity corona is abrupt, suddenly increasing the levels of electromagnetic interference and acoustic noise. Therefore, with ac conditions, as the applied voltage increases, the corona appears first during the negative semi period of the applied voltage.

**Figure 5:**
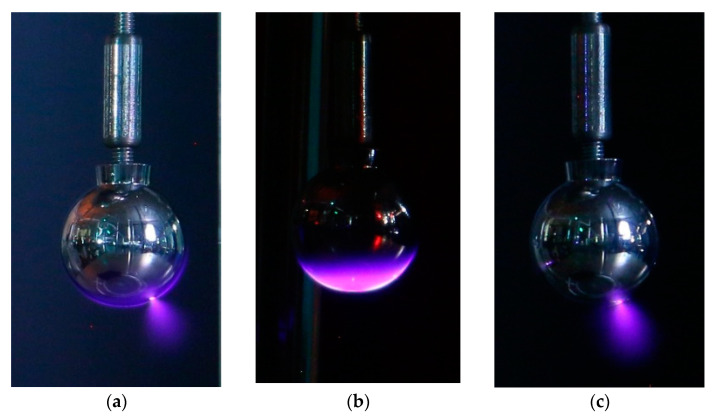
Sphere-plane gap. Photographs were taken with the DSLR camera. (**a**) Alternating current corona at 1 m. (**b**) Positive dc corona at 1 m. (**c**) Negative dc corona at 1 m.

**Figure 6:**
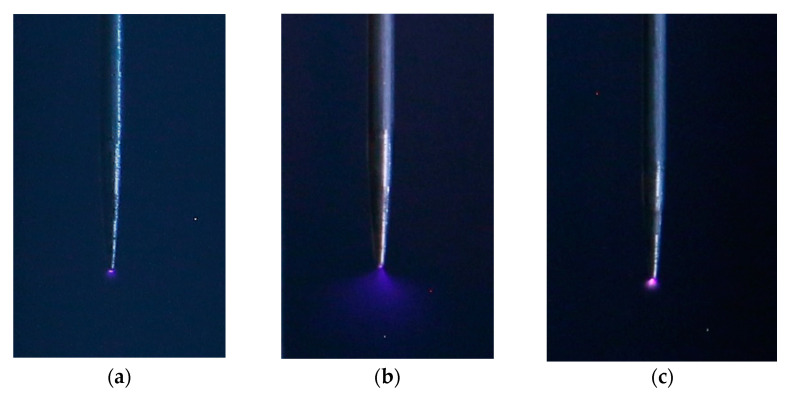
Needle-plane gap. Photographs were taken with the DSLR camera. (**a**) Alternating current corona at 1 m. (**b**) Positive dc corona at 1 m. (**c**) Negative dc corona at 1 m.

**Figure 7:**
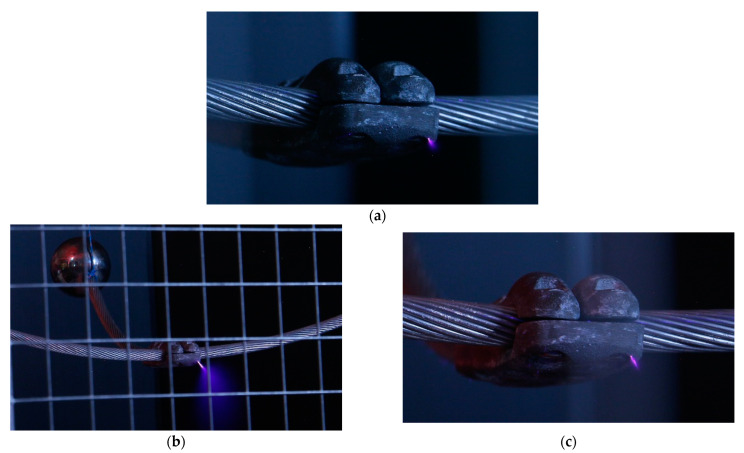
Substation connector. Photographs were taken with the DSLR camera. (**a**) Alternating current corona at 1 m. (**b**) Positive dc corona at 5 m. (**c**) Negative dc corona at 1 m.

The experimental that are results shown in this section have been obtained from the study of three geometries (sphere-plane, needle-plane, and connector-plane gaps) to generate corona discharges with three types of voltage, that is, 50 Hz alternating current, and positive and negative direct current supply. Figure 5, Figure 6 and Figure 7 show, respectively, photographs of the corona discharges of the three analyzed electrodes that were taken with the DSLR camera.

The objective of such tests is to compare the sensitivity of both cameras using different electrode geometries and different types of voltages (ac, positive and negative dc).

**Figure 8:**
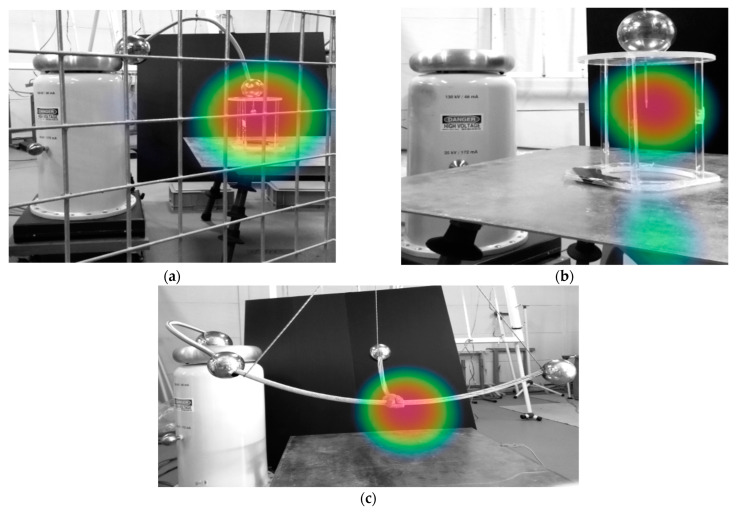
Photographs that were taken with the acoustic NL camera. (**a**) Sphere-plane gap at 2 m. (**b**) Needle-plane gap at 1 m. (**c**) Substation connector at 1 m.

Figure 8 shows some photographs that were taken by the acoustic NL camera of the different analyzed air gaps.

The images that are presented in Figure 7 and Figure 8 show that both DSLR and sonic cameras can locate corona discharges with great precision, although the DSLR camera provides a more precise location of corona sites as it directly detects the dim light that is emitted by the discharges. However, the DSLR camera requires long exposure photography in dark conditions, while the identification of corona sites by the sonic camera is almost instantaneous and can be done in daylight conditions.

As explained, the appearance of negative corona is gradual, as are the associated acoustic and electromagnetic emission levels, while generating very little electromagnetic interference and acoustic noise. In contrast, the appearance of the positive corona is abrupt and very energetic, often exhibiting higher CIV values compared to the negative corona.

Table 3 shows the experimental CIV values at different distances from the sphere (1-2-3-4-5-10 m) using the DSLR and sonic cameras. It is seen that both cameras have the same sensitivity for the sphere-plane gap geometry with 50 Hz ac and positive and negative dc supply. 

The results that are presented in Table 3, which were obtained with the sphere-plane gap, show the same CIV values with both cameras, regardless of the type of voltage that is applied. The sphere-plane gap is a simple geometry, and although the negative corona produces less light and acoustic emissions than the positive corona, could be heard directly with the ear.

Table 4 summarizes the experimental CIV values at different distances from the needle (1-2-3-4-5-10 m) using the DSLR and sonic cameras. The results show that both cameras have very similar sensitivity for the needle-plane gap geometry with 50 Hz ac and positive and negative dc supply.

The results that are presented in Table 4, which were obtained with the needle-plane gap, show almost the same CIV values with both cameras regardless of the type of voltage that was applied. Similar to the sphere-plane gap, the needle-plane gap is also a simple geometry, and from the results that are summarized in Table 3 and Table 4, it seems that the levels of acoustic and light emissions levels behave similarly with the different types of applied voltages.

Table 5 summarizes the experimental CIV values at different distances from the substation connector (1-2-3-4-5-10 m) using the DSLR and sonic cameras. 

The results that are presented in Table 5 show that when analyzing the corona discharges that were generated by a substation connector, which represents a complex geometry, both cameras still show very similar sensitivities with 50 Hz ac and positive and negative dc supply. However, the sonic camera is a bit less sensitive on ac and negative dc supply compared to the DSLR camera. The CIV value with ac supply corresponds to a negative corona, that is, it occurs in the negative semi period of the voltage wave, since when the voltage increases, the negative corona appears before the positive corona. Therefore, when analyzing complex geometries such as the substation connector, due to the low level of AN that is generated by negative corona discharges, the sonic camera is slightly less sensitive than the DSLR camera but has much faster response and can operate in daylight conditions. An important aspect to consider is that both cameras are sophisticated electronic devices, so the presence of dust, rain, fog, or liquids on the camera lens or on the microphone array is not desirable. 

**Figure 9:**
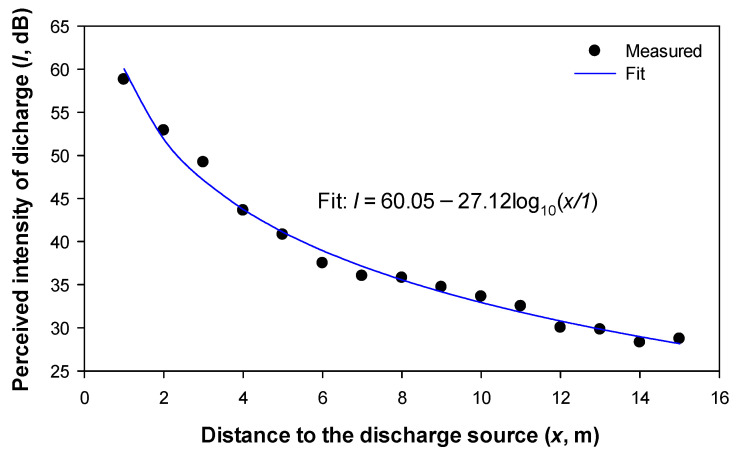
Logarithmic decay of the sound intensity of the discharges that were detected by the acoustic NL camera. Experimental points and logarithmic fit (*R*^2^ = 0.9891) that was measured with the sphere-plane gap with 50 Hz ac supply.

Sound intensity is known to decay logarithmically with the distance between the measurement point and the noise source. Assuming a perfectly spherical sound wave propagating away from the source uniformly in all directions, the intensity of sound reduces at a rate of 6 dB every time the distance to the source is doubled. This behavior corresponds to −20log_10_(*d*_2_/*d*_1_), where *d_i_* is the distance between the measurement point and the sound source. However, due to reflections on the walls and the ground plane, the expected results should differ somewhat from this law. To show this effect, Figure 9 summarizes the results that were obtained with the sonic camera using the sphere-plane gap with 50 Hz ac supply at different distances.

The results that are presented in Figure 9 show that instead of a decay rate of −20log_10_(*d*_2_/*d*_1_), the sound intensity decays as 60.05 − 27.12log_10_(*d*_2_/*d*_1_). This difference is mainly attributed to reflections with the ground, ceiling, walls, and surrounding objects. It is worth noting that although the theoretical decay rate is −20log_10_(*d*_2_/*d*_1_), the measured decay rate is higher due to the absorption of the walls, ceiling, ground, and surrounding objects, thus resulting in −27.12log_10_(*d*_2_/*d*_1_).

## 5. Conclusions

Corona discharges appear in high-voltage equipment and, in some conditions, in medium- and low-voltage equipment when the electric field strength exceeds a critical value. They generate harmful effects and different types of emissions, so their early detection and the location of discharge points are important to improve the design of said equipment and facilitate maintenance tasks. In this work, the performance of two cameras that allow detecting and locating the sources of corona discharges when there is a clear line of sight has been evaluated and compared; that is, an optical DSLR camera and a new sonic camera. In a previous work it was shown that in an unscreened laboratory, the DSLR camera is as sensitive as a commercial PD detector based on apparent charge, a standard method for PD detection. Therefore, due to its proven high sensitivity, the DSLR camera is taken as the reference. It is important to remark that conventional PD detection methods usually do not allow a clear and straightforward location of the exact discharge points, while the cameras that were analyzed in this work allow a clear location of the discharge sites.

The results that are presented in this work show that both cameras have a similar sensitivity regardless of the distance between the discharge sites and the sensor in the range of 1–10 m, although for complex electrodes with complex geometry the sonic camera is slightly less sensitive than the DSLR camera. The cost of the cameras is very different, since the sonic camera costs about 15 times more compared to the specific model of the DSLR camera that was used in this work. However, the main advantage of the sonic camera is that it is fully operational in daylight conditions and very easy to operate by an inexperienced user, while the DSLR camera requires complete or partial darkness. While the response of the sonic camera is almost instantaneous, the DSLR camera requires taking long-exposure photos to optimize its sensitivity, lasting at least 30 s to capture more photons that are generated by the corona. These characteristics are very important in selecting the most suitable camera for a specific application. The results that are presented in this work also show that the sonic camera allows a fast, simple, and sensitive detection and location of the source of the corona discharges even in a very incipient stage of the discharges.

## Figures and Tables

**Table 1:** Specifications of the DSLR camera.

Feature	Value/Description
Model	Canon EOS-70D
Sensor	CMOS APS-C sensor (22.5 × 15 mm)
Bandwidth	UV/visible
Resolution	20.2 Mpixels
Lens	18–135 mm

**Table 2:** Specifications of the sonic camera.

Feature	Value/Description
Model	NL Camera
Sensor	124 low-noise MEMS microphones
Bandwidth	2–65 kHz
Measurement distance	0.3–130 m
Snapshot resolution	800 × 480 pixels
Operating temperature	−10–50 °C

**Table 3:** CIV values that were obtained with the sphere-plane gap.

Feature	CIV Value (kV-RMS for ac, kV for dc)					
**ac (50 Hz)**	**1 m**	**2 m**	**3 m**	**4 m**	**5 m**	**10 m**
DSLR camera	44.3	44.3	44.3	44.3	44.3	44.3
Sonic camera	44.3	44.3	44.3	44.3	44.3	44.3
**Positive dc**	**1 m**	**2 m**	**3 m**	**4 m**	**5 m**	**10 m**
DSLR camera	71.5	71.5	71.5	71.5	71.5	71.5
Sonic camera	71.5	71.5	71.5	71.5	71.5	71.5
**Negative dc**	**1 m**	**2 m**	**3 m**	**4 m**	**5 m**	**10 m**
DSLR camera	−71.3	−71.3	−71.3	−71.3	−71.3	−71.3
Sonic camera	−71.3	−71.3	−71.3	−71.3	−71.3	−71.3

**Table 4:** CIV values that were obtained with the needle-plane gap.

Feature	CIV Value (kV-RMS for ac, kV for dc)					
**ac (50 Hz)**	**1 m**	**2 m**	**3 m**	**4 m**	**5 m**	**10 m**
DSLR camera	8.6	8.6	8.6	8.6	8.6	8.6
Sonic camera	8.6	8.6	8.6	8.6	8.6	9.2
**Positive dc**	**1 m**	**2 m**	**3 m**	**4 m**	**5 m**	**10 m**
DSLR camera	14.9	14.9	15.2	15.2	15.2	15.2
Sonic camera	15.2	15.2	15.2	15.2	15.2	15.2
**Negative dc**	**1 m**	**2 m**	**3 m**	**4 m**	**5 m**	**10 m**
DSLR camera	−14.8	−14.8	−14.8	−14.8	−14.8	−14.8
Sonic camera	−14.8	−14.8	−14.8	−14.8	−14.8	−14.8

**Table 5:** CIV values that were obtained with the substation connector.

Feature	CIV Value (kV-RMS for ac, kV for dc)					
**ac (50 Hz)**	**1 m**	**2 m**	**3 m**	**4 m**	**5 m**	**10 m**
DSLR camera	66.5	66.5	66.5	66.5	66.5	66.5
Sonic camera	67.9	67.9	67.9	67.9	67.9	69.0
**Positive dc**	**1 m**	**2 m**	**3 m**	**4 m**	**5 m**	**10 m**
DSLR camera	+111.5	+111.5	+111.5	+111.5	+111.5	+111.5
Sonic camera	+111.5	+111.5	+111.5	+111.5	+111.5	+111.5
**Negative dc**	**1 m**	**2 m**	**3 m**	**4 m**	**5 m**	**10 m**
DSLR camera	−95.0	−95.0	−95.0	−95.0	−95.0	−95.0
Sonic camera	−97.5	−97.5	−97.5	−97.5	−97.5	−97.5
